# Healthy lifestyle counseling, and barriers perceived by general practitioners in Poland

**DOI:** 10.3389/fpubh.2023.1256505

**Published:** 2023-09-27

**Authors:** Małgorzata Znyk, Dorota Kaleta

**Affiliations:** Department of Hygiene and Epidemiology, Medical University of Lodz, Lodz, Poland

**Keywords:** general practitioner (GP), primary care, healthy lifestyle, body mass index (BMI), counseling, Poland

## Abstract

**Introduction:**

The aim of the study was to determine the influence of the *personal factors*, such as body mass index (BMI), of general practitioners (GPs), and their counseling on weight management, healthy lifestyle, and their perceived barriers.

**Materials and methods:**

The cross-sectional study was conducted from January 2020 to December 2021 among 161 GPs in the city of Lodz.

**Results:**

Only 3.7% of physicians always advised their patients on diet and physical activity (*p* < 0.05). Most of the GPs (54%) provide counseling occasionally. GPs gave general advice more often to patients with chronic diseases than to patients who did not. The study showed that the chance of providing advice on eating habits or physical activity was significantly higher for the GPs who practiced physical activity (OR = 2.64; 95%Cl: 1.01–6.91, *p* < 0.05) and measured patient weight, height, and BMI (OR = 4.86; 95%Cl: 1.86–12.67, *p* < 0.001). GPs who were overweight (OR = 3.55; 95%Cl: 1.49–8.41, *p* < 0.01) and measured patient weight, height, and BMI (OR = 3.61; 95%Cl: 1.58–8.25, p < 0.01) were more likely to advise on nutrition or physical activity to “healthy” patients. Doctors who measured patient weight, height, and BMI advised patients with chronic diseases (OR = 6.45; 95%Cl: 2.54–16.34, *p* < 0.001). Over 40% of GPs believe that they are not effective in counseling. Lack of time turned out to be the main barrier to counseling for 73.3% of GPs, which was associated with heavy workload (>100 visits per week).

**Conclusion:**

As many GPs doubt their effectiveness, it is recommended that GPs attend more training activities regarding counseling. In addition, organizational changes are needed to reduce patient numbers, and financial incentives are needed to improve counseling and patient measurement.

## Introduction

Obesity is a public health problem, being a well-documented risk factor for many chronic diseases, such as cardiovascular disease, hypertension, coronary artery disease, type 2 diabetes, and some cancers (1, 2). In the 21st century, the world is facing an obesity pandemic (3). According to the World Health Organization, over a billion people worldwide are obese: 650 million adults, 340 million adolescents, and 39 million children (2). In 2019, 53% of people living in the European Union (EU) were overweight and 17% were obese. The highest percentage of overweight adults was recorded in Malta and Croatia (65%), and the lowest in Italy (46%) and France (47%). The percentage for Poland was 58% (2, 4). Currently, 65% of the world’s population lives in countries where overweight and obesity cause more deaths than underweight (3). This has significant direct and indirect costs on health care and social resources (2).

The development of the COVID-19 pandemic in 2020 and related lockdown had a clear influence on bodyweight in Poland (3). During the COVID-19 pandemic, 29% of Polish women and 28% of men noticed an increase in body weight. In 2020, 46% of Polish women and 64% of men were overweight, and 8% of women and 12% of men were obese (5, 6). Treatment of obesity presents a challenge for healthcare professionals (7), and the typical location for dietary and physical activity counseling is the primary care facility (8).

General practitioners (GPs) are in the best position to observe changes in body weight and to determine the environmental and psychological factors responsible for eating disorders (9). They are expected to help obese patients lose weight by counseling them on ways to change their health behaviors, and are often the only healthcare professionals the patient can see (1). To do this, it is important for the GP to build good relationships based on trust and cooperation with the patients, as they will feel more comfortable and may be more receptive to their recommendations, compared to a specialist (10).

Poland lacks a coordinated system for obesity treatment, and treatment is typically the responsibility of GPs and specialist physicians. If the doctor is unable or unwilling to treat obesity, the patient should be referred to another doctor who will (11). Most physicians initiate weight loss discussions once a patient is in the obese BMI category, rather than at a normal BMI or who are overweight (12). Doctors believe that many patients with obesity are ready to lose weight, and that weight advice provided by doctors can be successful (13).

Numerous studies show that the involvement of GPs in obesity treatment is positively perceived by patients and results in weight loss (1, 14). Advice from a GP increases patient self-efficacy, and supports their motivation and their efforts to lose weight (1, 15, 16). Such advice is highly valued by patients, and GP counseling has potential to reduce the incidence of preventable chronic diseases (17, 18).

General practitioners are expected to provide lifestyle counseling and preventive services to patients with chronic conditions. In addition, the personal lifestyle of the physicians themselves has an impact on patient care. However, few studies have examined whether such personal factors, such as body weight, influence the weight management practices of GPs toward patients in primary care (12, 19). There are also gaps in the literature on the measurements of body weight, height, and waist circumference performed by general practitioners.

Studies most often take into account such lifestyle habits as physical activity, diet, smoking, alcohol consumption, and hours of sleep (20, 21). However, they do not take into account the number of patients under care, completed university, and years of service, which may have a significant impact on the counseling provided.

Therefore, there is a need to better understand the roles played by the personal characteristics of the GP, such as weight, the number of patients under care, age, the university where they graduated and years of service, in their approach to obesity management in patients.

The aim of this study was to assess the frequency of dietary and physical activity counseling provided by GPs stratified by body mass index (BMI). Predictors of counseling among GPs were also determined.

It also examines the frequency at which GPs calculate BMI and measure body weight, and waist circumference in adult patients to assess the severity of obesity, and determines the course of action taken with patients diagnosed with overweight or obesity, i.e., whether advice is given or the fact is noted in the records.

## Materials and methods

### Study design and population

The cross-sectional study was conducted from January 2020 to December 2021 among GPs in the city of Lodz, Poland. In total, 433 GPs were currently practicing in the Lodz province as of December 31, 2019 (22). According to data received from the National Health Fund in 2020 and 2021, there were 211 primary healthcare entities in the city of Lodz at that time.

A number generator was used to randomly draw 120 numbers (23). From the list of 211 entities, 100 primary healthcare entities were selected using the first 100 randomly-drawn numbers. Of these, 11 refused to participate, and 11 clinics were selected from the next 11 generated numbers. In each of the 100 randomized subjects, two interviews with GPs were conducted.

In the morning on Monday or Wednesday and the afternoon on Tuesday or Friday, every second attending physician in the doctor’s office was randomly selected and asked to participate in the study. In case of disagreement, a third GP was selected. For inclusion, the GP had to be treating adult patients, who gave their written and voluntary consent to participate. The required sample size was calculated using the sample size calculator with a significance level of 0.05, fraction size of 0.5, maximum error of 5%, and population size of 433. About 200 participants were required. In total, 161 GPs joined the study, and 39 physicians refused to participate. 161 GPs completed the study. The participation rate was 80%.

The study was approved by the Bioethics Committee of the Medical University of Lodz (September 18, 2018; ref.: RNN/315/18/KE).

### Study variables

The research tool was an anonymous questionnaire containing standardized questions completed by the GP. The questionnaire was divided into sections. The first part contained information on the characteristics of the treated patients and information on the role of physician as a provider of a healthy lifestyle and health counseling. The study included elements of “a healthy lifestyle,” such as physical activity, diet, and weight. The second part concerned the barriers in the process of assessing, controlling, and managing the patients. The third part contained questions about the health status/health behaviors of primary care physicians. The questionnaire also included socio-demographic data (age, sex), information regarding the main place of medical practice, years of work, and number of patient visits during the routine working week.

The questionnaire was created by experts. The construction of the questionnaire (unambiguity, confidentiality, neutrality, and correctness of the scale of questions) was verified in an earlier large study (24) in order to ensure its reliability and credibility. Physicians’ questionnaires were anonymized. At the same time, a questionnaire survey was conducted among patients. The results were presented in the other articles (25, 26). Our research was anonymous, and thus the general practitioners could not be linked to their patients in any way.

The degree of counseling by the GP regarding a healthy diet/proper nutrition and physical activity was determined by the following questions: “How often do you recommend a healthy diet/proper nutrition,” “How often do you recommend physical activity” (Table 1). Advice included general and specific recommendations, such as eating different foods and spending time more actively. The response “never” indicated no advice given on eating habits, diet and physical activity in any appointment, “often” indicated that advice was given in 50% or more appointments, while “always” indicated advice given at each visit. Physicians who answered “often” or “always” were considered as providing advice on nutrition or physical activity in the univariate and multivariate logistic regression analysis (Table 2).

**Table 1:** Recommendations to change eating habits and physical activity by a GP in primary care patients.

Variable	Female *n = 119*			Male *n = 42*			Total *n = 161*		
	*n* (%)	95% Cl	*p* value	*n* (%)	95% Cl	*p* value	*n* (%)	95% Cl	*p* value
How often do you evaluate the patient’s diet and physical activity?									
Never	52 (43.7)	(34.79–52.61)	0.4369	16 (38.1)	(23.41–52.78)	0.3809	68 (42.3)	(34.61–49.87)	0.4223
Often	62 (52.1)	(43.13–61.08)	0.5210	25 (59.5)	(44.68–74.37)	0.5952	87 (54.0)	(46.33–61.74)	0.5404
Always	5 (4.2)	(0.60–7.81)	0.0420^*^	1 (2.4)	(−2.23–6.99)	0.0238^*^	6 (3.7)	(0.80–6.65)	0.0372^*^
How often do you recommend a healthy diet/proper nutrition?									
Never	17 (14.3)	(8.00–20.57)	0.1429	10 (23.8)	(10.93–36.69)	0.2381	27 (16.7)	(10.99–22.54)	0.1677
Often	75 (63.0)	(54.35–71.70)	0.6303	23 (54.8)	(39.71–69.81)	0.5476	98 (60.9)	(53.33–68.41)	0.6087
Always	27 (22.7)	(15.16–30.21)	0.2269	9 (21.4)	(9.01–33.84)	0.2143	36 (22.4)	(15.92–28.80)	0.2236
How often do you recommend physical activity?									
Never	22 (18.5)	(11.51–25.46)	0.1849	10 (23.8)	(10.93–36.69)	0.2381	32 (19.9)	(13.71–26.04)	0.1988
Often	69 (58.0)	(49.11–66.85)	0.5798	22 (52.4)	(37.28–67.49)	0.5238	91 (56.5)	(48.86–64.18)	0.5652
Always	28 (23.5)	(15.91–31.15)	0.2353	10 (23.8)	(10.93–36.69)	0.2381	38 (23.6)	(17.04–30.16)	0.2360

^*^p < 0.05.

**Table 2:** Correlates of advice provided by a GP.

Variables	Advice on changing eating habits or physical activity in patients (total) *N* = 161						Advice on changing eating habits or physical activity in patients with chronic diseases *N* = 161					Advice on changing eating habits or physical activity in “healthy” patients *N* = 161				
	Unadjusted model				Adjusted model		Unadjusted model			Adjusted model		Unadjusted model			Adjusted model	
	*N*	*n* = 134 (%)	OR	95% Cl	OR	95% Cl	*n* = 133 (%)	OR	95% Cl	OR	95% Cl	*n* = 103 (%)	OR	95% Cl	OR	95% Cl
Body mass index (BMI)																
< 25 kg/m^2^	93	79 (84.9)	1.00	Ref.			77 (82.8)	1.00	Ref.			53 (57.0)	1.00	Ref.	1.00	Ref.
≥ 25 to < 30 kg/m^2^	49	38 (77.6)	0.61	(0.25–1.48)			41 (83.7)	1.06	(0.42–2.72)			39 (79.6)	2.94	(1.31–6.64)^**^	3.55	(1.49–8.41) ^**^
≥ 30 kg/m^2^	19	17 (89.5)	1.51	(0.31–7.34)			15 (78.9)	0.78	(0.23–2.66)			11 (57.9)	1.04	(0.38–2.84)	0.89	(0.32–2.52)
Age (years)																
<40	55	46 (83.6)	1.02	(0.36–2.88)			47 (85.5)	1.70	(0.62–4.65)			38 (69.1)	0.99	(0.42–2.31)		
40–54	62	52 (83.9)	1.28	(0.45–3.67)			52 (83.9)	1.53	(0.56–4.22)			35 (56.5)	0.63	(0.28–1.42)		
55+	44	36 (81.8)	1.00	Ref.			34 (77.3)	1.00	Ref.			30 (68.2)	1.00	Ref.		
Sex																
Female	119	102 (85.7)	1.88	(0.78–4.54)			101 (84.9)	1.75	(0.73–4.21)			75 (63.0)	0.85	(0.40–1.80)		
Male	42	32 (76.2)	1.00	Ref.			32 (76.2)	1.00	Ref.			28 (66.7)	1.00	Ref.		
Number of chronic diseases																
0	59	48 (81.4)	1.00	Ref.			48 (81.4)	1.00	Ref.			35 (59.3)	1.00	Ref.		
1	45	40 (88.9)	1.83	(0.59–5.77)			40 (88.9)	1.83	(0.59–5.77)			33 (73.3)	1.89	(0.81–4.39)		
2	27	22 (81.5)	1.01	(0.31–3.28)			24 (88.9)	1.83	(0.47–7.28)			16 (59.3)	0.99	(0.39–2.54)		
≥3	30	24 (80.0)	0.92	(0.30–2.80)			21 (70.0)	0.53	(0.19–1.49)			19 (63.3)	1.18	(0.48–2.95)		
Diseases occurring in the family																
Diabetes mellitus																
Yes	77	65 (84.4)	1.18	(0.51–2.72)			62 (80.5)	0.76	(0.33–1.72)			47 (61.0)	0.78	(0.41–1.50)		
No	84	69 (82.1)	1.00	Ref.			71 (84.5)	1.00	Ref.			56 (66.7)	1.00	Ref.		
Coronary artery disease																
Yes	79	65 (82.3)	0.87	(0.38–2.01)			64 (81.0)	0.80	(0.35–1.83)			46 (58.2)	0.61	(0.32–1.17)		
No	82	69 (84.1)	1.00	Ref.			69 (84.1)	1.00	Ref.			57 (69.5)	1.00	Ref.		
Neoplastic disease																
Yes	80	68 (85.0)	1.29	(0.56–2.98)			69 (86.3)	1.67	(0.72–3.85)			51 (63.8)	0.98	(0.52–1.87)		
No	81	66 (81.5)	1.00	Ref.			64 (79.0)	1.00	Ref.			52 (64.2)	1.00	Ref.		
Subjective assessment of being overweight or obese																
Yes	39	31 (79.5)	0.71	(0.28–1.80)			30 (76.9)	0.61	(0.25–1.51)			25 (64.1)	1.01	(0.48–2.14)		
No	122	103 (84.4)	1.00	Ref.			103 (84.4)	1.00	Ref.			78 (63.9)	1.00	Ref.		
Weight loss intention																
Yes	76	66 (86.8)	1.65	(0.70–3.89)			65 (85.5)	1.48	(0.64–3.41)			50 (65.8)	1.16	(0.61–2.22)		
No	85	68 (80.0)	1.00	Ref.			68 (80.0)	1.00	Ref.			53 (62.4)	1.00	Ref.		
Physical activity																
Yes	126	111 (88.1)	3.86	(1.60–9.40)^**^	2.64	(1.01–6.91)^*^	106 (84.1)	1.57	(0.62–3.98)			81 (64.3)	1.06	(0.49–2.32)		
No	35	23 (65.7)	1.00	Ref.	1.00	Ref.	27 (77.1)	1.00	Ref.			22 (62.9)	1.00	Ref.		
Diet																
Yes	42	37 (88.1)	1.68	(0.59–4.80)			37 (88.1)	1.77	(0.62–5.05)			30 (71.4)	1.58	(0.73–3.40)		
No	119	97 (81.5)	1.00	Ref.			96 (80.7)	1.00	Ref.			73 (61.3)	1.00	Ref.		
Medical practice																
Private	104	85 (81.7)	0.73	(0.30–1.80)			86 (82.7)	1.02	(0.43–2.40)			71 (68.3)	1.68	(0.86–3.29)		
Public	57	49 (86.0)	1.00	Ref.			47 (82.5)	1.00	Ref.			32 (56.1)	1.00	Ref.		
Years of work																
<10	46	37 (80.4)	0.86	(0.33–2.23)			39 (84.8)	1.39	(0.52–3.75)			33 (71.7)	1.27	(0.57–2.85)		
10–20	40	35 (87.5)	1.47	(0.48–4.50)			34 (85.0)	1.42	(0.50–4.02)			20 (50.0)	0.50	(0.23–1.10)		
>20	75	62 (82.7)	1.00	Ref.			60 (80.0)	1.00	Ref.			50 (66.7)	1.00	Ref.		
Number of patient visits during the routine working week																
≤100	59	49 (83.1)	0.98	(0.41–2.31)			47 (79.7)	0.73	(0.32–1.68)			35 (59.3)	0.73	(0.38–1.42)		
>100	102	85 (83.3)	1.00	Ref.			86 (84.3)	1.00	Ref.			68 (66.7)	1.00	Ref.		
Lack of time as a barrier to counseling																
Yes	118	97 (82.2)	0.75	(0.28–2.01)			101 (85.6)	2.04	(0.86–4.84)			74 (62.7)	0.81	(0.39–1.71)		
No	43	37 (86.0)	1.00	Ref.			32 (74.4)	1.00	Ref.			29 (67.4)	1.00	Ref.		
Appropriate training to provide counseling on nutrition, physical activity, and weight management																
Yes	122	106 (86.9)	2.60	(1.08–6.28) ^*^	1.59	(0.59–4.23)	106 (86.9)	2.94	(1.24–7.00)^*^	1.79	(0.68–4.69)	83 (68.0)	2.02	(0.96–4.24)		
No	39	28 (71.8)	1.00	Ref.	1.00	Ref.	27 (69.2)	1.00	Ref.	1.00	Ref.	20 (51.3)	1.00	Ref.		
Making measurements of body weight, height, BMI																
Yes	125	113 (90.4)	6.73	(2.74–16.52)^***^	4.86	(1.86–12.67) ^***^	113 (90.4)	7.53	(3.08–18.43)^***^	6.45	(2.54–16.34)^***^	87 (69.6)	2.86	(1.33–6.16)^**^	3.61	(1.58–8.25)^**^
No	36	21 (75.0)	1.00	Ref.	1.00	Ref.	20 (55.6)	1.00	Ref.	1.00	Ref.	16 (44.4)	1.00	Ref.	1.00	Ref.

^*^p < 0.05, ^**^p < 0.01, and ^***^p < 0.001; Fully-adjusted model, including all statistically significant characteristics. Ref, Reference; CI, Confidence interval.

The questionnaire also surveyed the frequency of weight measurements on scales, weight declared by the patient, calculation of BMI, and measurement of height, and waist circumference by the GP: (1) at each routine visit, (2) once a year, (3) if clinically indicated, and (4) never (Table 3).

**Table 3:** Frequency of measurement of body weight and other variables by a GP in primary care patients.

Variable	Female *n* = 119			Male *n* = 42			Total *n* = 161		
	*n* (%)	95% Cl	*p* value	*n* (%)	95% Cl	*p* value	*n* (%)	95% Cl	*p* value
Body weight measured on the scale									
At each routine visit	7 (5.9)	(1.65–10.11)	0.0588	5 (11.9)	(2.11–21.70)	0.1190	12 (7.5)	(3.40–11.51)	0.0745
Once a year	28 (23.5)	(15.91–31.15)	0.2353	6 (14.3)	(3.70–24.87)	0.1429	34 (21.1)	(14.81–27.42)	0.2111
If clinically indicated	74 (62.2)	(53.47–70.90)	0.6218	27 (64.3)	(49.79–78.78)	0.6429	101 (62.7)	(55.26–70.20)	0.6273
Never	10 (8.4)	(3.42–13.39)	0.0840	4 (9.5)	(0.65–18.40)	0.0952	14 (8.7)	(4.34–13.05)	0.0869
The patient’s declared body weight									
At each routine visit	23 (19.3)	(12.23–26.42)	0.1933	2 (4.8)	(−1.68–11.20)	0.0476	25 (15.5)	(9.93–21.12)	0.1553
Once a year	34 (28.6)	(20.45–36.69)	0.2857	13 (30.9)	(16.97–44.93)	0.3095	47 (29.2)	(22.17–36.22)	0.2919
If clinically indicated	51 (42.9)	(33.97–51.75)	0.4286	23 (54.8)	(39.71–69.81)	0.5476	74 (46.0)	(38.26–53.66)	0.4596
Never	11 (9.2)	(4.04–14.45)	0.0924	4 (9.5)	(0.65–18.40)	0.0952	15 (9.3)	(4.83–13.81)	0.0932
Body mass index (BMI)									
At each routine visit	15 (12.6)	(6.64–18.57)	0.1261	4 (9.5)	(0.65–18.40)	0.0952	19 (11.8)	(6.82–16.78)	0.1180
Once a year	35 (29.4)	(21.23–37.60)	0.2941	10 (23.8)	(10.93–36.69)	0.2381	45 (28.0)	(21.02–34.88)	0.2795
If clinically indicated	62 (52.1)	(43.13–61.08)	0.5210	25 (59.5)	(44.68–74.37)	0.5952	87 (54.0)	(46.34–61.74)	0.5403
Never	7 (5.9)	(1.65–10.11)	0.0588	3 (7.2)	(−0.65–14.93)	0.0714	10 (6.2)	(2.48–9.94)	0.0621
Waist measurement									
At each routine visit	7 (5.9)	(1.65–10.11)	0.0588	1 (2.4)	(−2.23–6.99)	0.0238^*^	8 (5.0)	(1.61–8.33)	0.0496^*^
Once a year	20 (16.8)	(10.09–23.53)	0.1681	7 (16.7)	(5.40–27.94)	0.1667	27 (16.8)	(11.00–22.54)	0.1677
If clinically indicated	70 (58.8)	(49.98–67.67)	0.5882	25 (59.5)	(44.68–74.37)	0.5952	95 (59.0)	(51.41–66.60)	0.5901
Never	22 (18.5)	(11.51–25.46)	0.1849	9 (21.4)	(9.02–33.84)	0.2143	31 (19.2)	(13.16–25.35)	0.1925
Height measurement									
At each routine visit	14 (11.8)	(5.98–17.55)	0.1176	4 (9.5)	(0.65–18.40)	0.0952	18 (11.2)	(6.31–16.05)	0.1118
Once a year	29 (24.3)	(16.66–32.08)	0.2437	8 (19.1)	(7.17–30.92)	0.1905	37 (23.0)	(16.48–29.48)	0.2298
If clinically indicated	66 (55.5)	(46.53–64.39)	0.5546	24 (57.1)	(42.18–72.11)	0.5714	90 (55.9)	(48.23–63.57)	0.5590
Never	10 (8.4)	(3.42–13.39)	0.0840	6 (14.3)	(3.70–24.87)	0.1429	16 (9.9)	(5.32–14.56)	0.0994

^*^p < 0.05.

The age and gender of the GPs was recorded. The doctors were also asked to provide their height (m) and weight (kg), which allowed the BMI (kg/m^2^) to be calculated thus: weight (kg)/height squared (m^2^) (27).

Physicians were divided into three groups according to BMI: <25 kg/m^2^ (normal), ≥ 25 to <30 kg/m^2^ (overweight), and ≥ 30 kg/m^2^ (obese). The characteristics of the studied population are presented in Table 4.

**Table 4:** The characteristics of the studied population of general practitioners (GPs).

Characteristics	Total *n = 161*	%
Age (years)		
<40	55	34.2
40–54	62	38.5
55+	44	27.3
Sex		
Male	42	26.1
Female	119	73.9
Body mass index (BMI)		
<25 kg/m^2^	93	57.8
≥25 to <30 kg/m^2^	49	30.4
≥30 kg/m^2^	19	11.8
Number of chronic diseases		
0	59	36.6
1	45	28.0
2	27	16.8
≥3	30	18.6
Diseases occurring in the family		
Diabetes mellitus	77	47.8
Coronary artery disease	79	49.1
Neoplastic disease	80	49.7
Subjective assessment of being overweight or obese		
Yes	39	24.2
No	122	75.8
Weight loss intention		
Yes	76	47.2
No	85	52.8
Physical activity		
Yes	126	78.3
No	35	21.7
Diet		
Yes	42	26.1
No	119	73.9
Medical practice		
Private	104	64.6
Public	57	35.4
Years of work		
<10	46	28.6
10–20	40	24.8
>20	75	46.6
Number of patient visits during the routine working week		
≤100	59	36.6
>100	102	63.4
Lack of time as a barrier to counseling		
Yes	118	73.3
No	43	26.7
Appropriate training to provide counseling on nutrition, physical activity, and weight management		
Yes	122	75.8
No	39	24.2
Making measurements of body weight, height, and BMI		
Yes	125	77.6
No	36	22.4

The GPs were asked about the occurrence of any pre-chronic diseases related to overweight and obesity, such as hypertension, coronary artery disease, type 2 diabetes, chronic obstructive pulmonary disease, asthma, and others. According to the answers, they were divided into four groups: (1) no diseases, (2) one disease, (3) two diseases, and (4) three or more diseases. The GPs were also asked about their subjective assessment of being overweight and obese, and their intention to lose weight.

The survey also examined the lifestyle characteristics of the GPs, such as fruit and vegetable consumption and physical activity. Respondents consuming an average of 400 g of vegetables and fruit in their daily diet, i.e., in at least five portions, were considered healthy eaters (28). Those who performed 150–300 min of moderate-intensity physical activity per week or 75–150 min of vigorous-intensity physical activity were considered physically active subjects (29).

In addition, the GPs were surveyed regarding the number of patient visits during a routine working week, i.e., (group 1) ≤ 100 and (group 2) > 100, as well as their years of service, i.e., (group 1) < 10 or (group 2) 10–20 or (group 3) > 20, and the nature of their medical practice, i.e., (group 1) private or (group 2) public.

The doctors were also asked whether lack of time was a barrier to counseling (Yes, No), and whether they had received appropriate training to provide counseling on nutrition, physical activity, and weight management (Yes, No).

The questionnaire also included questions about the doctor’s treatment of an obese patient: (1) “Systematically observes/tracks patients’ behavior or other measures of progress regarding diet, physical activity, and body weight, and records them in the documentation”; (2) “Prescribes a patient a drug treatment aimed at losing weight”; (3) “Referral of the patient to obesity treatment surgery”; (4) “Referring to another health professional or offering participation in programs conducted outside of practice,” with the possible answers Yes or No (Table 5).

**Table 5:** The procedure implemented by GPs in patients with overweight and obese.

Variable	Female *n =* 119 (%)	Male *n =* 42 (%)	Total *n =* 161 (%)
Systematically observes/ tracks patients’ behavior or other measures of progress regarding diet, physical activity, and body weight, and records in the documentation			
Yes	108 (90.8)	36 (85.7)	144 (89.4)
No	11 (9.2)	6 (14.3)	17 (10.6)
Prescribes a patient a drug treatment aimed at losing weight			
Yes	75 (63.0)	20 (47.6)	95 (59.0)
No	44 (37.0)	22 (52.4)	66 (41.0)
Referral of the patient to obesity treatment surgery			
Yes	65 (54.6)	23 (54.8)	88 (54.7)
No	54 (45.4)	19 (45.2)	73 (45.3)
Referring to another health professional or offering participation in programs conducted outside of his practice			
Yes	103 (86.6)	36 (85.7)	139 (86.3)
No	16 (13.4)	6 (14.3)	22 (13.7)

The questionnaire also included the following statements: “A physician is obliged to provide counseling focusing on a healthy lifestyle among the patients,” “I have sufficient knowledge and skills to advise patients on healthy lifestyle,” “I am effective in helping my patients to lead a healthy lifestyle,” and “The healthy lifestyle counseling will be more effective if the physician himself/herself follows health recommendations.” Five responses were possible for each statement: “I strongly agree,” “I tend to agree,” “I do not know,” “I tend to disagree,” and “I strongly disagree.” The first two options (“I strongly agree,” and “I tend to agree”) were considered positive (“Yes”), and the rest (“I do not know,” “I tend to disagree” and “I strongly disagree”) negative (“No”; Table 6).

**Table 6:** Opinions about the role of a GP as a healthy lifestyle provider.

Opinion	Female *n* = 119 (%)	Male *n* = 42 (%)	Total *n* = 161 (%)
A physician is obliged to provide counseling focusing on a healthy lifestyle among the patients			
Yes	109 (91.6)	36 (85.7)	145 (90.1)
No	10 (8.4)	6 (14.3)	16 (9.9)
I have sufficient knowledge and skills to advise patients on healthy lifestyle			
Yes	93 (78.2)	29 (69.0)	122 (75.8)
No	26 (21.8)	13 (31.0)	39 (24.2)
I am effective in helping my patients to lead a healthy lifestyle			
Yes	72 (60.5)	22 (52.4)	94 (58.4)
No	47 (39.5)	20 (47.6)	67 (41.6)
Healthy lifestyle counseling will be more effective if a physician is following health himself recommends			
Yes	112 (94.1)	34 (81.0)	146 (90.7)
No	7 (5.9)	8 (19.0)	15 (9.3)

### Statistical analysis

The descriptive statistics and a distribution of examined variables were calculated as numbers and percentages. The distribution of categorical variables is represented by frequency and proportion with 95% confidence intervals. Categorical variables were compared using the chi-square test for independent samples. *p* < 0.05 was considered statistically significant. Elements of logistic regression analysis were used to describe the obtained results.

Univariate and multivariate logistic regression analyses were performed to identify predictors of GP preventive action; results were presented as odds ratios (OR) and 95% confidence intervals (95% CI). Variables with *p* < 0.1 from the univariate analysis were included in the multivariate model. STATISTICA version 13.3 (StatSoft, licensed by the Medical University of Lodz) was used for the analysis.

## Results

### Characteristics of the studied population

The characteristics of the study population of GPs are presented in Table 4. Of the study group, 73.9% were women and 26.1% were men. The largest group of respondents were GPs aged 40–54 (38.5%). Of 161 surveyed GPs of the city of Lodz, 30.4% were overweight and 11.8% were obese. In the subjective assessment, 89.5% of obese physicians and 40.8% of overweight physicians reported having excessive BMI. Of these 47.2% declared an intention to lose weight. Among the doctors, 28.0% reported one chronic disease, 16.8% two chronic diseases, and 18.6% had three or more chronic diseases. The percentage of chronic diseases occurring in GPs is presented in Table 7.

**Table 7:** Chronic diseases in general practitioners (GPs).

Variables	Total *n = 161*	%
Abnormal body mass index BMI		
Yes	39	24.2
No	122	75.8
Abnormal lipid profile		
Yes	32	19.9
No	129	80.1
Hypertension		
Yes	34	21.1
No	127	78.9
Eating disorders such as anorexia or bulimia		
Yes	2	1.2
No	159	98.8
Asthma		
Yes	6	3.7
No	155	96.3
Type II diabetes		
Yes	12	7.5
No	149	92.5
Coronary artery disease		
Yes	3	1.9
No	158	98.1
Cancer		
Yes	5	3.1
No	156	96.9
Arthritis		
Yes	7	4.3
No	154	95.7
Sleep apnea		
Yes	4	2.5
No	157	97.5
Chronic obstructive pulmonary disease		
Yes	3	1.9
No	158	98.1
Back pain		
Yes	61	37.9
No	100	62.1
Hypothyroidism		
Yes	7	4.3
No	154	95.7

In addition, 78.3% of the respondents reported engaging in 150–300 min a week of moderate intensity physical activity of 75–150 min of high intensity, and 26.1% consumed an average of 400 g of vegetables and fruit in their daily diet in at least five portions (Table 4).

The data also indicate that 64.6% of GPs are employees of private medical practices and 35.4% of public medical practices, and that 46.6% had been working for at least 20 years. Furthermore, 63.4% reported more than 100 patient visits during a routine working week.

Lack of time was reported as the main barrier to counseling among 73.3% of GPs, and 75.8% indicated that they had received appropriate training to provide counseling on nutrition, physical activity, and weight management. In addition, 77.6% measured of the body weight, height, and BMI of their patients.

### Advice on nutrition and physical activity

The data indicate that 57.7% of GPs evaluate the physical activity and diet of the patient; however, only 3.7% of physicians indicated they “always” advised their patients on diet and physical activity (*p* < 0.05); including 2.4% of male physicians (*p* < 0.05) and 4.2% of female physicians (*p* < 0.05; Table 1). In total, 54.0% of physicians “often” assessed diet and physical activity.

Otherwise, 22.4% of GPs “always” recommended a healthy diet/proper nutrition to their patients, and 23.6% always recommended physical activity, while 60.9% “often” recommended a healthy diet/proper nutrition to their patients, and 56.5% physical activity.

The personal characteristics of GPs were compared with their likelihood of providing counseling using logistic regression analyses. The strength of the relationship was represented by odds ratio (OR) and 95% confidence interval (CI). The results of the univariate and multivariate logistic regression analyses and health services are presented in Table 2.

The GPs were more likely to give general advice to patients with chronic diseases than to those without.

Variables that were found to be significant in the univariate logistic regression analysis were considered in the multivariate logistic regression analysis. The multivariate analysis found that GPs who practiced physical activity (OR = 2.64; 95%Cl: 1.01–6.91, *p* < 0.05) and measured patient weight, height and BMI (OR = 4.86; 95%Cl: 1.86–12.67, *p* < 0,001) were more likely to provide advice on eating habits or physical activity than those who do not. Physicians who measured weight, height and BMI (OR = 6.45; 95%Cl: 2.54–16.34, *p* < 0.001) were more likely to advise on diet or physical activity in patients with chronic diseases. GPs who were overweight were more likely to give advice on nutrition or physical activity in “healthy” patients (OR = 3.55, 95%Cl: 1.49–8.41, *p* < 0.01), and to measure patient weight, height, and BMI (OR = 3.61; 95%Cl: 1.58–8.25, p < 0.01) compared to normal-weight or obese physicians, who did not perform any measurements.

### Body weight measurement

In total, 7.5% of the surveyed physicians indicated that they measure the body weight of their patients at each routine visit, and 62.7% indicated that body weight was measured in clinical settings (Table 3). 5% of primary care physicians measured waist circumference at each routine visit (*p* < 0.05) including 2.4% of male physicians (*p* < 0.05).

Similarly, based on clinical indications, 46.0% of GPs assessed the body weight declared by the patient, 54.0% calculated the BMI, 59.0% measured the waist circumference, and 55.9% the height.

### The procedure implemented by GPs in patients with overweight or obesity

Table 5 presents the procedure implemented by GPs in patients with overweight or obesity. In total, 89.4% of GPs reported systematically tracking patient behavior regarding diet, physical activity, and body weight, while 59% prescribe a drug treatment aimed at losing weight in patients with overweight or obesity, particularly among female physicians (63%). In addition, 86.3% of GPs refer the patient to another healthcare professional or offer participation in programs conducted outside of their practice.

### The role of a physician as a healthy lifestyle provider

Table 6 presents possible opinions about the role of a GP as a healthy lifestyle provider. Ninety percent of the GPs believed that the doctor was obliged to provide patients with healthy lifestyle counseling, and 75.8% thought that they had sufficient knowledge and skills to do so. More than half of the GPs believed that they were effective in helping patients to lead healthy lifestyles (58.4%).

Nearly all GPs (90.7%) believed that healthy lifestyle counseling would be more effective if the GPs themselves followed health recommendations. Female physicians were more likely to advocate that the physician can advise on a healthy lifestyle.

## Discussion

This study was the first during the COVID-19 pandemic to assess the extent to which GPs monitor and assess the health behaviors of overweight and obese patients and provide appropriate advice in this respect. It also examined the prevalence of overweight and obesity among the GPs themselves, and assessed various predictors that may influence the attitudes of the GPs toward dietary and physical activity counseling among their patients.

General practitioners should strive to implement activities related to a healthy lifestyle among their patients. Our survey results show that 90% of GPs believe they have a duty to advise patients on a healthy lifestyle. As many as 75% of them believe that they have sufficient knowledge to do so.

In our study, 30.4% of physicians were overweight and 11.8% were obese, and a significantly low percentage of physicians aged 40–54 (33.3%) and aged 55 and over (19.4%) had a normal BMI. Other results were obtained in the study of Alnasiri et al. (19) where 50% of the study participants were overweight while 16.1% were obese, and a significantly low percentage of physicians aged 40–54 (23.1%) and under 40 (39.3%) had a normal BMI.

The likelihood of initiating discussions about weight loss depended on the physician’s BMI: while physicians with a normal body weight were more likely to discuss body weight than those who were overweight or obese, the differences were not statistically significant, except for advice given to healthy patients by overweight doctors. In our study, 79.6% of overweight GPs advised healthy patients on diet and physical activity (*p* < 0.01).

Previous studies have shown that physicians with either a normal BMI or overweight/obese advised their patients to exercise regularly and make some dietary changes to control obesity (19). In the study by Alnasiri et al. (19), 95% of overweight GPs advised patients to exercise and change their diet.

About 80% of GPs in our study advised their patients on diet or physical activity; however, only one in five indicated that they “always” provide advice on diet and physical activity. Similar results were obtained by Alnasiri et al. (19), where over 90% of the surveyed physicians indicated advising their patients on exercise and diet for weight loss, and by Al-Shammari et al. (30), where 70.8% of physicians advised their patients on eating habits, and 76.9% on physical activity. Also, two-thirds of physicians in the United States reported frequently holding consultations (18). Another study found that less than 50% of surveyed physicians always give specific advice on diet, physical activity or weight management (31).

General practitioners more often gave advice on physical activity than on diet or weight control (31). About 70% of GPs in a German study (32) said that they routinely give at least half of their patient’s brief consultations on dietary modifications, and more than 90% in an Australian study reported proactively talking to their patients about nutrition (33).

More than 90% of the GPs in our study felt that diet and exercise advice would be more effective if the GPs themselves followed health recommendations. More precisely, 11% did not assess diet, physical activity or BMI when examining patients. Such patients do not receive appropriate advice regarding healthy lifestyle.

Physicians who exercise regularly and follow a healthy diet have been shown to be more likely to address BMI reduction through diet and exercise (34, 35). This is confirmed by our present findings. GPs with a normal BMI were more likely to provide advice on nutrition and physical activity when they engaged in physical activity themselves. GPs are also aware of the important role played by primary health care in health education and highlight the need for its greater participation in promoting a healthy lifestyle (36). A similar positive attitude by physicians toward overweight and obesity counseling was also noted in the present study. GPs have been found to generally demonstrate a positive attitude toward the role of nutrition in the prevention and treatment of diseases (37, 38). Most GPs in Canada, the United States and Australia agreed it would improve patient outcomes in chronic disease (39–42). More than 90% of Lebanese GPs agreed that nutritional counseling effectively influenced patient behavior (43). Dumic et al. (44) showed that one-third of the surveyed Croatian physicians expressed a positive attitude toward nutrition and nutritional care, and the majority declared that they would provide nutritional care in their daily practice. Studies conducted in Germany and the United States confirm pretty positive attitudes regarding the role of physicians in health promotion, disease prevention, and obesity counseling (32, 45, 46). However, other studies conducted in Croatia (44, 47), Saudi Arabia (48), Australia and New Zealand (49) suggest that GPs show little interest in nutritional care, which may be a consequence of insufficient nutritional knowledge and lack of appropriate education and training (48).

Interventions focusing on the assessment and promotion of physical activity are also conducted by GPs in many countries (50–52); indeed, 80% of Americans and 77% of Canadians see a GP at least once a year, and during these visits physical activity is recommended (53, 54). Several studies have documented negative attitudes by physicians regarding counseling (1, 12, 55, 56); in particular, they have reported doubts as to whether it will influence patient behavior (12, 55) and a feeling that obesity is the responsibility of the patient (12, 55).

Despite the opportunities and benefits that dietary and physical activity counseling interventions can bring, they are provided relatively infrequently (57–61). Nutritional counseling is still underexploited. GPs highlight a number of barriers that hinder the practice of nutritional counseling (62) and physical activity counseling, such as inadequate weight counseling training, lack of time, and a need to prioritize comorbidities (12, 13). Similarly, almost two-thirds of GPs in our study indicated lack of time as the main barrier to counseling, despite having appropriate training to provide counseling (75.8%), as well as the large number of patients during a routine working week.

Other studies also indicate that little time is spent on diet and physical activity counseling by physicians in primary health care (63), with some reporting only 8% of time spent with the primary care physician being used to discuss overweight and obesity (63, 64). Similarly, some previous studies indicate the main reasons for poor lifestyle interventions to be insufficient time and lack of reimbursement (65). Inadequate time was noted by 61% GPs as the major barrier to counseling regarding physical activity (35).

Dietary and physical activity advice is integral to all weight loss consultations and should ideally be imparted by a dietician or a nutritionist (66). However, in Poland, dietitians are often unavailable in primary health care and the duty of lifestyle counseling rests with the GP. New legal provisions in force from October 1, 2022, intended to provide patients with dietary consultations and educational advice as part of coordinated care, require the GP to cooperate with specialist doctors and a dietitian (67, 68).

The predictors of healthy lifestyle counseling identified in our study, *viz.* sex, age, health status, physical activity, BMI, weight loss intention, working years, and the number of patient visits during a routine working week, may be important considerations when designing appropriate strategies aimed at improving public health.

Our study also examined the frequency with which GPs calculate BMI and measure weight and waist circumference in adult patients. According to recommendations in Poland, BMI should be calculated once a year for each adult (3).

Anthropometric measurements (body weight, height, and waist circumference) should be performed during the first meeting and during routine visits in patients with excessive body weight, or when this is suspected, together with any related complications (3). Our findings indicate that annual BMI measurements and calculations are rarely performed. Typically, doctors measure and calculate BMI when clinically indicated. Only one in 10 GPs in our study calculate patient BMI at each routine visit.

Other studies have found that Hite et al. (69) report that 91% of physicians routinely calculate BMI, and only 61% routinely discuss BMI. Other studies have found that approximately half of the studied GPs, *viz.* 49% (31) and 60% (30), reported regularly recording BMI. Our study shows that the studied GPs undertake counseling with their patients, and do not just note this fact in the patient’s documentation. The GP systematically observes the patient’s behavior regarding diet, physical activity, and body weight (89.4% GPs). Overweight or obese patients receive pharmacological treatment to lose weight and, if necessary, are referred to another health specialist. 59% of GPs prescribe drug treatment for weight loss and 86.3% refer patients to another health specialist.

Our study found no differences in overweight and obesity counseling between private and public medical practices. While many service providers feel comfortable providing exercise and dietary advice (69), more financial resources are spent on treatment than prevention in Poland.

The study has limitations. It was cross-sectional, being conducted at a single-time point, which makes it impossible to observe changes over longer periods of time. In addition, due to the COVID-19 pandemic of the time, access to GPs was more limited due to government restrictions. Also, the questionnaire did not include a question about the purpose of the patient’s visit; however, regardless of the reason for the visit, a GP should always notice overweight and obesity and give advice on a healthy lifestyle. Furthermore, a lack of association was noted in the multivariate analyses, possibly due to the small sample size, the anonymous nature of the study prevented patients from being linked with particular GPs, and all assessments were carried out only among GPs and did not include the opinions of patients. It should also be noted that the observed patterns of physicians may reflect the situation in urban areas; however, physicians are trained in the same way and their approach to risk factor assessment, counseling, and treatment should be independent of the workplace.

The study also has certain strengths. Most importantly, it is one of the first to examine the relationship between the personal characteristics of GPs (including BMI) and their readiness, and ability to provide counseling on overweight and obesity in primary care patients. It is certainly the first to do so in Poland during the COVID-19 pandemic. Few such studies have been prepared in other countries.

Nutritional and physical activity counseling provided by GPs was assessed using self-reported questionnaire data.

The results may play an important role in the development of health programs aimed at reducing overweight and obesity in Poland and elsewhere. Tackling obesity should be linked to an overall strategy to combat chronic non-communicable diseases and health promotion efforts. Specific interventions in this regard may include: introducing early recognition and management of overweight and obesity in primary health care, providing training on obesity prevention for family doctors, and increasing reimbursement for this type of health services. Further research on the influence of a GP on the health behavior of patients in primary health care and the identification of predictors of healthy lifestyle counseling is recommended.

## Conclusion

Our findings suggest that variation in physician BMI had an effect on their practice and counseling of obesity care. The likelihood of measuring body weight, height, and BMI by GPs were associated with a greater chance of giving advice on diet or physical activity. In addition, a high proportion of the studied physicians were found to be overweight and obese themselves.

Physicians should be properly trained so that the scope and frequency of advice provided should not be influenced by their individual characteristics, but only by their patient’s health condition. GPs are “role models” and hence should serve as good examples for patients. A doctor with a normal BMI will be more credible for a patient than a doctor who is overweight or obese, which can help make weight loss advice more effective.

The is a need for more practical guidelines, greater community involvement and more comprehensive training among primary care physicians to improve obesity treatment in Poland. The latter are particularly important as a significant percentage of doctors have little opportunity to improve their knowledge and skills in this area and doubt their effectiveness. A health policy that helps overcome barriers and create a stimulating environment for GPs to effectively implement nutrition and physical activity counseling strategies is key to improving the quality and quantity of counseling.

In addition, organizational changes are needed to reduce the number of patients admitted to reducing time constraints, and financial incentives are needed to improve counseling and patient measurement. GPs should be encouraged to advise healthy patients on how to prevent overweight and obesity, not only those already affected by the problem. Our findings reinforce the need for the development of educational strategies to support dietary and physical activity intervention among primary care physicians.

## Data availability statement

The original contributions presented in the study are included in the article/supplementary material, further inquiries can be directed to the corresponding author.

## Author contributions

MZ: Conceptualization, Data curation, Formal analysis, Investigation, Methodology, Project administration, Software, Validation, Writing – original draft, Writing – review & editing. DK: Supervision, Writing – review & editing.

## Abbreviations

GP: General practitioner
BMI: Body mass index
WHO: World Health Organization
EU: European union
OR: Odds ratio
CI: Confidence interval
US: United States
COVID-19: Coronavirus disease 2019
SARS-CoV-2: Severe acute respiratory syndrome coronavirus 2

## References

[1] Price CH Seviour R Twells L. The role of the primary care physician in obesity prevention. Can J Diabetes. (2013) 37:S269–9. doi: 10.1016/j.jcjd.2013.03.271

[2] World Health Organization World obesity day 2022-accelerating action to stop obesity. (2022). Available at: https://www.who.int/news/item/04-03-2022-world-obesity-day-2022accelerating-action-to-stop-obesity (Accessed May 11, 2023).

[3] Ostrowska L Bogdański P Mamcarz A. Obesity and its complications. Practical Diagnostic and Treatment Recommendations; PZWL, Warsaw, Poland (2021).

[4] Eurostat (2023). Over half of adults in the EU are overweight. Available at: https://ec.europa.eu/eurostat/web/products-eurostat-news/-/ddn-20210721-2 (Accessed May 11, 2023).

[5] Pinkas J. Current Public Health Challenges. Warsaw, Poland: PZWL, Medical Publishing House (2021).

[6] Wojtyniak B Goryński P. Health situation of the polish population and its conditions 2020; National Institute of Public Health National Institute of Hygiene: Warsaw, Poland (2020).

[7] Piccinini-Vallis H. Diagnosis, and management of obesity: a survey of general practitioners’ awareness of and familiarity with the 2006 Canadian clinical practice guidelines. Can J Diabetes. (2011) 35:170–15. doi: 10.1016/S1499-2671(11)52119-7

[8] Wattanapisit A Tuangratananon T Thanamee S. Physical activity counseling in primary care and family medicine residency training: a systematic review. BMC Med Educ. (2018) 18:159. doi: 10.1186/s12909-018-1268-1, PMID: 29970092 PMC6029015

[9] Olszanecka-Glinianowicz M Godycki-Cwirko M Lukas W Mastalerz-Migas A Tomasik T Tomiak E. Principles of overweight and obesity management in the practice of a family physician. Guidelines of the College of Family Physicians in Poland, the polish Society of Family Medicine and the polish Society for Obesity Research. Fam Doc Spec. (2017) 3:1–52.

[10] Kringos DS Boerma WG Hutchinson A van der Zee J Groenewegen PP. The breadth of primary care: a systematic literature review of its core dimensions. BMC Health Serv Res. (2010) 10:65. doi: 10.1186/1472-6963-10-65, PMID: 20226084 PMC2848652

[11] Olszanecka-Glinianowicz M Dudek D Filipiak KJ Krzystanek M Markuszewski L Ruchała M. Guidelines treatment of overweight and obesity during and after the pandemic. Let’s not wait for complications to develop—new guidelines for doctors. Arterial Hypertens. (2020) 24:93–105. doi: 10.5603/AH.a2020.0019 33740809

[12] Bleich SN Bennett WL Gudzune KA Cooper LA. Impact of physician BMI on obesity care and beliefs. Obesity (Silver Spring). (2012) 20:999–1005. doi: 10.1038/oby.2011.402, PMID: 22262162 PMC3645927

[13] Forman-Hoffman V Little A Wahls T. Barriers to obesity management: a pilot study of primary care clinicians. BMC Fam Pract. (2006) 7:35. doi: 10.1186/1471-2296-7-35, PMID: 16756673 PMC1525170

[14] Bennett WL Wang NY Gudzune KA Dalcin AT Bleich SN Appel LJ. Satisfaction with primary care provider involvement is associated with greater weight loss: results from the practice-based POWER trial. Patient Educ Couns. (2015) 98:1099–105. doi: 10.1016/j.pec.2015.05.006 26026649 PMC4546866

[15] Kant AK Miner P. Physician advice about being overweight: association with self-reported weight loss, dietary, and physical activity behaviors of US adolescents in the National Health and nutrition examination survey, 1999–2002. Pediatrics. (2007) 119:e142–7. doi: 10.1542/peds.2006-1116, PMID: 17200241

[16] Kurz D McCrea-Robertson S Nelson-Brantley H Befort C. Rural engagement in primary care for optimizing weight reduction (REPOWER): a mixed methods study of patient perceptions. Patient Educ Couns. (2022) 105:2371–81. doi: 10.1016/j.pec.2021.11.028, PMID: 34865892

[17] Maderuelo-Fernandez JA Recio-Rodríguez JI Patino-Alonso MC Pérez-Arechaederra D Rodriguez-Sanchez E Gomez-Marcos MA. Effectiveness of interventions applicable to primary health care settings to promote Mediterranean diet or healthy eating adherence in adults: a systematic review. Prev Med. (2015) 76:S39–55. doi: 10.1016/j.ypmed.2014.12.011 25524613

[18] Nair D Hart A. Family Physicians’ perspectives on their weight loss nutrition counseling in a high obesity prevalence area. J Am Board Fam Med. (2018) 31:522–8. doi: 10.3122/jabfm.2018.04.170467, PMID: 29986977

[19] Alnasiri ASA Alruwaili NM. Influence of physicians’ BMI on counseling practice for obesity in primary health care clinics in Aljouf region, Saudi Arabia. A cross-sectional study. J Fam Med Prim Care. (2021) 10:4143–6. doi: 10.4103/jfmpc.jfmpc_700_21, PMID: 35136780 PMC8797116

[20] Mahler L Sebo P Favrod-Coune T Moussa A Cohidon C Broers B. The prevalence of five lifestyle risk factors in primary care physicians: a cross-sectional study in Switzerland. Prev Med Rep. (2022) 26:101740. doi: 10.1016/j.pmedr.2022.101740, PMID: 35251911 PMC8889261

[21] Borgan SM Jassim GA Marhoon ZA Ibrahim MH. The lifestyle habits and wellbeing of physicians in Bahrain: a cross-sectional study. BMC Public Health. (2015) 15:655. doi: 10.1186/s12889-015-1969-x, PMID: 26170021 PMC4499902

[22] Statistical Yearbook of the Province of Lodz (2020). Statistical Office in Łódź (2020). Available at: https://lodz.stat.gov.pl/publikacje-i-foldery/roczniki-statystyczne/rocznik-statystyczny-wojewodztwa-lodzkiego-2020,6,22.html (Accessed May 11, 2023).

[23] Number Generator. Random number generator-random numbers. (2023). Available at: https://generatorliczb.pl/ (Accessed May 11, 2023).

[24] Znyk M Polańska K Wojtysiak P Szulc M Bąk-Romaniszyn L Makowiec-Dąbrowska T. Predictors of counselling related to a healthy lifestyle carried out by a general practitioner. Int J Environ Res Public Health. (2019) 16:4475. doi: 10.3390/ijerph16224475, PMID: 31739411 PMC6888170

[25] Znyk M Zajdel R Kaleta D. Consulting obese and overweight patients for nutrition and physical activity in primary healthcare in Poland. Int J Environ Res Public Health. (2022) 19:7694. doi: 10.3390/ijerph19137694, PMID: 35805379 PMC9265845

[26] Znyk M Wężyk-Caba I Kaleta D. The frequency of tobacco smoking and E-cigarettes use among primary health care patients—the association between anti-tobacco interventions and smoking in Poland. Int J Environ Res Public Health. (2022) 19:11584. doi: 10.3390/ijerph191811584, PMID: 36141847 PMC9517004

[27] Body Mass Index. Healthy lifestyle - WHO recommendations. (2023). Available at: https://www.who.int/europe/news-room/fact-sheets/item/a-healthy-lifestyle---who-recommendations (Accessed May 11, 2023).

[28] World Health Organization (2023). The World Health Organization recommends eating fruits and vegetables. Available at: https://krokdozdrowia.com/swiatowa-organizacja-zdrowia-zaleca-jedzenie-owocow-i-warzyw/ (Accessed May 21, 2023).

[29] World Health Organization WHO Guidelines on Physical Activity and sedentary behavior. (2020). Available at: https://ncez.pzh.gov.pl/aktywnosc-fizyczna/nowe-zalecenia-who-dotyczace-aktywnosci-fizycznej/ (Accessed May 21, 2023).

[30] Al-Shammari YF. Attitudes and practices of primary care physicians in the Management of Overweight and Obesity in eastern Saudi Arabia. Int J Health Sci. (2014) 8:151–8. doi: 10.12816/0006081, PMID: 25246882 PMC4166987

[31] Smith AW Borowski LA Liu B Galuska DA Signore C Klabunde C. U.S. primary care physicians’ diet-, physical activity-, and weight-related care of adult patients. Am J Prev Med. (2011) 41:33–42. doi: 10.1016/j.amepre.2011.03.017, PMID: 21665061 PMC3142674

[32] Schneider S Diehl K Bock C Herr RM Mayer M Görig T. Modifying health behavior to prevent cardiovascular diseases: a Nationwide survey among German primary care physicians. Int J Environ Res Public Health. (2014) 11:4218–32. doi: 10.3390/ijerph110404218, PMID: 24739770 PMC4025039

[33] Crowley J O’Connell S Kavka A Ball L Nowson CA. Australian general practitioners’ views regarding providing nutrition care: results of a national survey. Public Health. (2016) 140:7–13. doi: 10.1016/j.puhe.2016.08.013, PMID: 27692586

[34] Spencer EH Frank E Elon LK Hertzberg VS Serdula MK Galuska DA. Predictors of nutrition counseling behaviors and attitudes in US medical students. Am J Clin Nutr. (2006) 84:655–62. doi: 10.1093/ajcn/84.3.655 16960182

[35] Abramson S Stein J Schaufele M Frates E Rogan S. Personal exercise habits and counseling practices of primary care physicians: a national survey. Clin J Sport Med. (2000) 10:40–8. doi: 10.1097/00042752-200001000-00008, PMID: 10695849

[36] Stefanowicz A Kulik TB Pacian J Żołnierczuk-Kieliszek D Skorzyńska H. The role of primary health care facilities in the implementation of cancer prevention in the opinion of family doctors. Med Og Nauk Zdr. (2013) 19:168–72.

[37] Al-Gassimi O Shah HBU Sendi R Ezmeirlly HA Ball L Bakarman MA. Nutrition competence of primary care physicians in Saudi Arabia: a cross-sectional study. BMJ Open. (2020) 10:e033443. doi: 10.1136/bmjopen-2019-033443, PMID: 31911521 PMC6955539

[38] Rurik I Torzsa P Ilyés I Szigethy E Halmy E Iski G. Primary care obesity Management in Hungary: evaluation of the knowledge, practice and attitudes of family physicians. BMC Fam Pract. (2013) 14:156. doi: 10.1186/1471-2296-14-156, PMID: 24138355 PMC3853764

[39] Wynn K Trudeau JD Taunton K Gowans M Scott I. Nutrition in primary care: current practices, attitudes, and barriers. Can Fam Physician. (2010) 56:e109–16. PMID: 20228290 PMC2837706

[40] Aggarwal M Singh-Ospina N Kazory A Joseph I Zaidi Z Ataya A. The mismatch of nutrition and lifestyle beliefs and actions among physicians: a wake-up call. Am J Lifestyle Med. (2019) 14:304–15. doi: 10.1177/1559827619883603, PMID: 32477033 PMC7232894

[41] Fitzgerald JD Andrade JM Curl SL Smith EB Torna E Nelson DS. Development of nutrition counselling resources for family medicine using the knowledge to action framework. Fam Pract. (2021) 38:32–7. doi: 10.1093/fampra/cmaa020, PMID: 32255188

[42] Mitchell LJ Mac Donald-Wicks L Capra S. Nutrition advice in general practice: the role of general practitioners and practice nurses. Aust J Prim Health. (2011) 17:202–8. doi: 10.1071/PY10101, PMID: 21645478

[43] Hseiki RA Osman MH El-Jarrah RT Hamadeh GN Lakkis NA. Knowledge, attitude and practice of Lebanese primary care physicians in nutrition counseling: a self-reported survey. Prim Health Care Res Dev. (2017) 18:629–34. doi: 10.1017/S1463423617000330, PMID: 28606212

[44] Dumic A Miskulin I Pavlovic N Cacic Kenjeric D Orkic Z Miskulin M. Attitudes toward nutrition care among general practitioners in Croatia. J Clin Med. (2018) 7:60. doi: 10.3390/jcm7040060, PMID: 29561751 PMC5920434

[45] Diehl K Gansefort D Herr RM Görig T Bock C Mayer M. Physician gender and lifestyle counselling to prevent cardiovascular disease: a Nationwide representative study. J Public Health Res. (2015) 4:534. doi: 10.4081/jphr.2015.534, PMID: 26425495 PMC4568424

[46] Petrin C Kahan S Turner M Gallagher C Dietz WH. Current attitudes and practices of obesity counselling by health care providers. Obes Res Clin Pract. (2017) 11:352–9. doi: 10.1016/j.orcp.2016.08.005, PMID: 27569863

[47] Dumic A Miskulin I Matic Licanin M Mujkic A Cacic Kenjeric D Miskulin M. Nutrition counselling practices among general practitioners in Croatia. Int J Environ Res Public Health. (2017) 14:1499. doi: 10.3390/ijerph14121499, PMID: 29207514 PMC5750917

[48] Alkhaldy AA. Nutritional knowledge and self-reported nutritional practice against malnutrition among physicians in Jeddah, Saudi Arabia. Health. (2019) 7:149. doi: 10.3390/healthcare7040149, PMID: 31752253 PMC6955858

[49] Ball LE Hughes RM Leveritt MD. Nutrition in general practice: role and workforce preparation expectations of medical educators. Aust J Prim Health. (2010) 16:304–10. doi: 10.1071/PY10014, PMID: 21138698

[50] Huijg JM Gebhardt WA Verheijden MW Phillips EM. Factors influencing primary health care professionals’ physical activity promotion behaviors: a systematic review. Int J Behav Med. (2015) 22:32–50. doi: 10.1007/s12529-014-9398-2, PMID: 24788314

[51] Grogg KA Giacobbi PR Kelley GA. Physical activity assessment and promotion in clinical settings in the United States: a scoping review. Am J Health Promot. (2022) 36:714–37. doi: 10.1177/08901171211051840, PMID: 35224998

[52] Hartman SJ Risica PM Gans KM Marcus BH Eaton CB. Tailored weight loss intervention in obese adults within primary care practice: rationale, design, and methods of choose to lose. Contemp Clin Trials. (2014) 38:409–19. doi: 10.1016/j.cct.2014.06.001, PMID: 24937016 PMC4151311

[53] Warburton DE Charlesworth S Ivey A Nettlefold L Bredin SS. A systematic review of the evidence for Canada’s physical activity guidelines for adults. Int J Behav Nutr Phys Act. (2010) 7:39. doi: 10.1186/1479-5868-7-39, PMID: 20459783 PMC3583166

[54] Gagliardi AR Abdallah F Faulkner G Ciliska D Hicks A. Factors contributing to the effectiveness of physical activity counselling in primary care: a realist systematic review. Patient Educ Couns. (2015) 98:412–9. doi: 10.1016/j.pec.2014.11.020, PMID: 25499578

[55] Bleich SN Bandara S Bennett WL Cooper LA Gudzune KA. Impact of non-physician health professionals’ BMI on obesity care and beliefs. Obesity (Silver Spring). (2014) 22:2476–80. doi: 10.1002/oby.20881, PMID: 25185506 PMC4236247

[56] Huizinga MM Cooper LA Bleich SN Clark JM Beach MC. Physician respect for patients with obesity. J Gen Intern Med. (2009) 24:1236–9. doi: 10.1007/s11606-009-1104-8 19763700 PMC2771236

[57] Ahmed NU Delgado M Saxena A. Trends and disparities in the prevalence of physicians’ counseling on diet and nutrition among the U.S. adult population, 2000–2011. Prev Med. (2016) 89:70–5. doi: 10.1016/j.ypmed.2016.05.014, PMID: 27196147

[58] Tremblay MS Warburton DER Janssen I Paterson DH Latimer AE Rhodes RE. New Canadian physical activity guidelines. Appl Physiol Nutr Metab. (2011) 36:36–46. doi: 10.1139/H11-009 21326376

[59] Williams NH. “the wise, for cure, on exercise depend”: physical activity interventions in primary care in Wales. Br J Sports Med. (2009) 43:106–8. doi: 10.1136/bjsm.2008.051458 18669546

[60] van der Wardt V di Lorito C Viniol A. Promoting physical activity in primary care: a systematic review and meta-analysis. Br J Gen Pract. (2021) 71:e399–405. doi: 10.3399/BJGP.2020.0817, PMID: 33824160 PMC8049206

[61] Bock C Diehm C Schneider S. Physical activity promotion in primary health care: results from a German physician survey. Eur J Gen Pract. (2012) 18:86–91. doi: 10.3109/13814788.2012.675504, PMID: 22548286

[62] Vrkatić A Grujičić M Jovičić-Bata J Novaković B. Nutritional knowledge, confidence, attitudes towards nutritional care and nutrition counselling practice among general practitioners. Health. (2022) 10:2222. doi: 10.3390/healthcare10112222, PMID: 36360563 PMC9691229

[63] Abshire DA Gibbs S McManus C Caldwell T Cox A. Interest, resources, and preferences for weight loss programs among primary care patients with obesity. Patient Educ Couns. (2020) 103:1846–9. doi: 10.1016/j.pec.2020.04.003, PMID: 32331826 PMC7423734

[64] Tsai AG Abbo ED Ogden LG. The time burden of overweight and obesity in primary care. BMC Health Serv Res. (2011) 11:191. doi: 10.1186/1472-6963-11-191, PMID: 21846407 PMC3175444

[65] Lambe B Collins C. A qualitative study of lifestyle counselling in general practice in Ireland. Fam Pract. (2010) 27:219–23. doi: 10.1093/fampra/cmp086 20008029

[66] Chopra S Malhotra A Ranjan P Vikram NK Singh N. Lifestyle-related advice in the management of obesity: a step-wise approach. J Educ Health Promot. (2020) 9:239. doi: 10.4103/jehp.jehp_216_20, PMID: 33209931 PMC7652086

[67] Minister of Health (2023). Regulation of the Minister of Health of February 15, 2021 on guaranteed benefits in the field of primary health care (Dz.U. z 2021, poz. 540). Available at: https://www.infor.pl/akt-prawny/DZU.2021.084.0000540/rozporzadzenie-ministra-zdrowia-w-sprawie-swiadczen-gwarantowanych-z-zakresu-podstawowej-opieki-zdrowotnej.html (Accessed May 21, 2023).

[68] Minister of Health (2023). Regulation of the Minister of Health of September 19, 2022 amending the regulation on guaranteed benefits in the field of primary health care. (Dz.U. 2022, poz. 1965). Available at: https://isap.sejm.gov.pl/isap.nsf/download.xsp/WDU20220001965/O/D20221965.pdf (Accessed May 21, 2023).

[69] Hite A Victorson D Elue R Plunkett BA. An exploration of barriers facing physicians in diagnosing and treating obesity. Am J Health Promot. (2019) 33:217–24. doi: 10.1177/0890117118784227, PMID: 29986601

